# Photo-electrons unveil topological transitions in graphene-like systems

**DOI:** 10.1038/srep36577

**Published:** 2016-11-11

**Authors:** Lucila Peralta Gavensky, Gonzalo Usaj, C. A. Balseiro

**Affiliations:** 1 Centro Atómico Bariloche and Instituto Balseiro, Comisión Nacional de Energa Atómica, 8400 Bariloche, Argentina; 2 Consejo Nacional de Investigaciones Cientficas y Técnicas (CONICET), Argentina

## Abstract

The topological structure of the wavefunctions of particles in periodic potentials is characterized by the Berry curvature Ω_*kn*_ whose integral on the Brillouin zone is a topological invariant known as the Chern number. The bulk-boundary correspondence states that these numbers define the number of edge or surface topologically protected states. It is then of primary interest to find experimental techniques able to measure the Berry curvature. However, up to now, there are no spectroscopic experiments that proved to be capable to obtain information on Ω_*kn*_ to distinguish different topological structures of the *bulk* wavefunctions of semiconducting materials. Based on experimental results of the dipolar matrix elements for graphene, here we show that ARPES experiments with the appropriate x-ray energies and polarization can unambiguously detect changes of the Chern numbers in dynamically driven graphene and graphene-like materials opening new routes towards the experimental study of topological properties of condensed matter systems.

Topology plays a central role in defining the structure of the ground state of condensed matter systems, the nature of the excitations and their response to external probes^1^,^2^,^3^,^4^. For particles in periodic potentials, like electrons in solids, cold atoms systems or photonic crystals, the topology of the Bloch wavefunctions is related to the geometric or Berry phase acquired by the particle as it moves along a closed path in reciprocal space^5^. Within a given energy band, these phases are characterized by the Berry curvature (Ω_***k**n*_) whose integral over the Brillouin zone (BZ) is a topological invariant, the Chern number.

According to the bulk-boundary correspondence principle, the Chern numbers determine the unbalance in the number of chiral edge (or surface) states^1^. Experimentally, it has been easier to study the effects of a non-trivial topology, *i.e*. the emergence of such chiral edge states, rather than its origin: the structure of the Bloch wavefunctions across the whole BZ. In fact, transport and spectroscopic experiments provide direct evidence on the existence of the edge states^6^,^7^,^8^,^9^. Extracting information on Ω_*kn*_ and its integral in the BZ as a measure of topology in condensed matter systems has been more elusive.

Since it is Ω_***k**n*_ what encodes all the information on topology and non-local effects it is natural to look for ways of obtaining direct information about this quantity—even in systems with trivial topology Ω_***k**n*_ is associated with anomalous velocities^5^,^10^,^11^ and may lead to non-local conductances and unconventional (valley) Hall effects. Ultra-cold atoms in optical lattices offer a unique playground for the study of topological band structures^12^ and during the last years a number of experiments focused on the study of different structures, including hexagonal lattices with bosonic and fermionic atoms. In particular, recent experiments were able to obtain a complete tomographic image of the Berry curvature of a Bloch band^13^. No such experiments, that require a fast switching off of the confining (lattice) potentials, are possible in solids.

The question then arises as to what experiments could give direct information on the topological structure of the Bloch wavefunctions in condensed matter systems. The high resolution angle resolved photoemission spectroscopy (ARPES) has proven to be a powerful tool to measure the dispersion relation of low energy bands^7^, the band structure of dynamically driven systems (Floquet spectrum)^14^,^15^, quasiparticle lifetimes and even the chiral nature of the electronic states in graphene systems^16^. In the latter case, ARPES experiments show that the intensity patterns have an angular dependence that give direct information of the Berry’s phase. This is due to the fact that graphene’s wavefunctions are spinors corresponding to the pseudo-spin associated with the two sublattices of the hexagonal structure. Then, close to the Dirac points, the pseudo-spin is parallel to the crystal momentum leading to a nontrivial Berry phase of *π*. Similar results are obtained in bilayer graphene where the winding angle is 2*π*. However, neither the band structure nor the Berry phase around the Dirac cones provide enough information to fully characterize the topological structure of the bands.

In what follows we show that using a pump and probe setup in graphene and graphene-like systems, photo-electrons can unveil topological phase transitions, *i.e*. they can unambiguously detect changes in the Chern numbers. On the one hand this is possible due to the structure of the dipole matrix elements linked to the excitation of the electrons at the *π*-bands of graphene. On the other hand, although Chern numbers involve the Berry curvature of all *k*-points in the BZ, the largest contribution comes from two *hot spots*—the Dirac points. As we show below, detailed analysis of the intensity of photo-electrons coming from the corners of the BZ gives the required information to identify topological transitions.

Non-trivial topologies may be generated by external magnetic fields, spin-orbit coupling or by dynamically driving a system with external time dependent fields^17^,^18^,^19^,^20^,^21^,^22^. The latter creates a new class of topological insulators known as Floquet Topological Insulators (FTI). In what follows we consider such a case.

Pump and probe experiments consist in coupling the system to an electromagnetic pump pulse followed by a short photo-exciting ARPES pulse. We consider spatially homogeneous pump pulses of circularly polarized light of frequency Ω. Typical duration of the pump pulse is *δt*_pump _~_ _250 fs. The photo-excitation due to the probe pulse occurs during the pumping time, being its duration 1/Ω < *δt*_probe _~_ _*δt*_pump_ and its polarization either linear or circular.

It is instructive to start our analysis with a simple model of a gaped graphene-like system with a mass term. The pump pulse is described by a vector potential ***A***(*t*) so that, for the crystal momentum ***k*** close to the *K* or *K*′ points of the BZ, the Hamiltonian reads


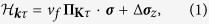


where 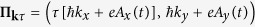, *τ* = ± corresponds to the *K* or *K*′ Dirac points, respectively, and the components of ***σ*** are the Pauli matrices—in our notation the up (down) pseudo-spin corresponds to the *A* (*B*) sublattice. This model describes the band structure of graphene (with Δ = 0) and of silicene or germanene where the mass gap Δ can be induced by an external electric field^23^ as well as a variety of 2D materials and artificial structures^24^.

Before including the full time dependence of the pump pulse, we consider a circularly polarized monochromatic radiation described by ***A***(*t*) = Re(***A***_0_ *e*^*i*Ω*t*^) with 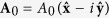. The time dependent Schrödinger equation can be solved in the frame of the Floquet theory^25^,^26^,^27^,^28^. For states with wavevector close to the Dirac points, and to the lowest order in the field amplitude *A*_0_, the system can be described by the following Floquet Hamiltonian


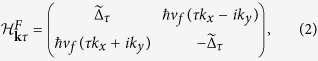


with


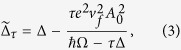


and Floquet quasi-energies 
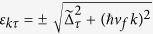
. This solution shows that at the *K* point (*τ* = +) the gap decreases as the field amplitude increases, it closes at a critical value 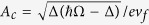 and increases again for *A*_0_ > *A*_*c*_. On the other hand, the gap at *K*′ increases monotonously^29^. Reversing the sense of rotation of the electromagnetic field changes the Dirac point at which the gap closes. This phenomena, known as band inversion, is accompanied by a change of the nature of the Floquet wavefunctions at the corners of the BZ. While for *A*_0_ < *A*_*c*_ the wavefunctions of the conduction band for both cones at ***k*** = **0** are localized on the *A* sublattice (*i*.*e*. their pseudo-spin is up), for *A*_0_ > *A*_*c*_ the wavefunction at *K* lies on sublattice *B* (down pseudo-spin) as shown in Fig. 1.

The band inversion with the closing of the gap at the critical field amplitude signals a topological phase transition^29^,^30^. Indeed the Berry curvature of the Floquet states around *K* and *K*′ is given by


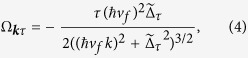


and gives a contribution to the Chern number of the (Floquet) valence band 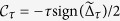
5. Hence, to this order in the field amplitude, 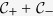 changes from 0 for *A*_0_ < *A*_*c*_ to 1 for *A*_0_ > *A*_*c*_. When considering the full tight-binding Hamiltonian of graphene, it can be shown that in undoped nanoribbons the phase having 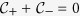 behaves as a (normal) insulator. In this phase the gap is preserved with non protected edge states laying close to the bottom and top of the energy gap. Conversely, in the phase where 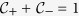 the gap is bridged by topologicaly protected chiral edge states and the system becomes a TI^31^.

We are now in position to address the problem of how the intensity and angular dependence of the ARPES distinguishes the two different topological phases. The photo-excitation process is described by the Hamiltonian


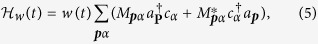


where *w*(*t*) describes the time profile of the probe pulse, 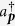 creates a photo-electron with total momentum ***p*** and *c*_*α*_ annihilates an electron at the sample with quantum numbers *α*. Assuming that the probe pulse *w*(*t*) acts in the time interval [*t*_0_, *t*_1_], the total photo-electron distribution obtained after the probe is given by^16^,^32^,^33^


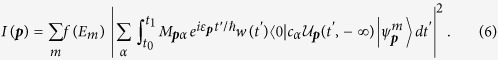


here *ε*_***p***_ is the energy of the photo-electron, 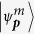 is a state of the system in equilibrium with energy *E*_*m*_, 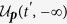 is the time evolution operator including the effect of the pump pulse and *f* (*E*) is the Fermi-Dirac distribution. Only the evolution of the bulk states due to the pump perturbation is taken into account, neglecting effects of coupling of the radiation field with the final high-energy states. This effect has already been addressed in recent work^15^, giving rise to Volkov states. It has been pointed out that the dressing of the free-electron states can be neglected if the polarization of the pumping field is on the plane of the surface of the irradiated material. Since in our case we only consider normal incidence for the pumping field, it is correct to ignore it. The structure of the dipolar matrix elements *M*_***p**α*_ has been discussed in ref. 16 for a probe pulse described by a vector potential with a polarization vector given by 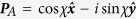. Using as a complete basis the eigenstates of the unperturbed system (*α* = ***k***, *τ*, ± where ± indicate the valence and conduction bands, respectively) and setting 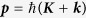 or 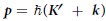 we have 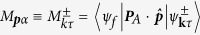 with *ψ*_*f*_ the wavefunction of the final photo-electron state and 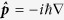
—the choice of parametrization for the probe polarization allows for the use of symmetry arguments to determine *ψ*_*f*_^16^. These matrix elements are given in terms of the dipole transition matrix elements 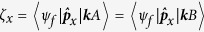 and 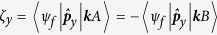 for the *x* and *y* components of the probe pulse respectively (see Supplementary Information). In the expressions above, |***k**A*〉 and |***k**B*〉 are the Bloch wavefunctions of the *A* and *B* sublattices, respectively.

Recent experiments^16^ showed that for graphene the ratio *ζ*_*y*_/*ζ*_*x*_ = *λe*^*iβ*^ depends on the frequency of the photo-emitting probe pulse: *λ* is on the order of one and 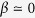 while for high energies (~30 eV) while 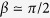 for lower energies (~20 eV). It is worth to emphasize that these particular values of *β* depend on the final state *ψ*_*f*_ and hence on the choice of symmetry of the polarization of the probe pulse, whose principal axes are always along the *x* and *y* directions. In the former case the momentum distribution of the photo-electrons gives valuable information on the Berry phase and has been analyzed in detail in ref. 16 and in subsequent works in the absence of the pump perturbation^34^,^35^. In this case a simple calculation gives the following photo-electron distribution due to electrons with quantum numbers ***k***, *τ*, ±,


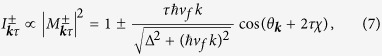


with *θ*_***k***_ = arctan(*k*_*y*_/*k*_*x*_).

Photons with different polarization selectively excite electrons in the BZ generating a marked dichroism. This is reflected in the angular dependence of constant energy maps of 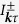 close to the *K* point. The angular dependence of the photo-electron distribution highlights the chiral nature of the initial states and gives direct information of the winding phase *θ*_***k***_. Similar results are obtained with a pump pulse as shown in Fig. 1(d). To lowest order in the pump amplitude, the photo-electron intensities are given by Eq. (7) where now Δ is to be replaced by 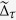. Then the pump changes the band structure as suggested by the lowest order Floquet Hamiltonian [cf. Eq. (2)] and the closing of the mass gap at *K* can be observed. However, under these conditions (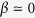) the ARPES spectrum cannot distinguish the two different topological phases. In fact, the *β* = 0 photo-electron distribution 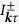 is independent of the sign of the mass term, which means that the intensity pattern remains invariant under a change in the orientation of the pseudo-spin along the *z* axis. In this case, although the ARPES can detect the closing and reopening of the gap at one of the Dirac points as the amplitude of the pump pulse increases, this cannot be unambiguously assigned to a band inversion. In particular, in graphene where Δ = 0 the gaps at the two Dirac point are identical and the photo-electron intensities are insensitive to the sign of 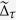.

However, when 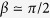, a situation experimentally observed for 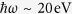, the ARPES spectrum changes at the critical amplitude of the pump pulse allowing for a clear identification of the topology of the Floquet bands. Before presenting the numerical results we may get some insight into the problem by evaluating the photo-electron distribution using again the lowest order Floquet Hamiltonian. For *λ* = 1 and *β* = *π*/2 this approach gives


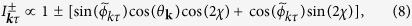


with 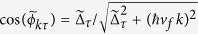 and 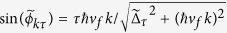. This simple result makes apparent that the ARPES spectrum for non-linear polarization of the probe (*χ* ≠ 0 or *π*/2) depends on the sign of the mass term. Consequently, the topological transition is manifested as a change in the amplitude of the photo-electron intensities showing different behaviors at the *K* and *K*′ cones. Under this choice of parameters it is possible to generate a photo-electron distribution with purely A or B character, *i.e*. to selectively photo-emit states with different pseudo-spin polarization along the *z* axis. Equation (8) also shows that the dichroism depends on the helicity of the probe. Defining the dichroism factor *D* as the normalized maximum angular variation of the photoemission intensity along a constant energy curve we obtain 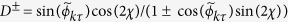. For a circularly polarized probe pulse (*χ* = *π*/4) we have that *D*^±^ = 0 and the information on the Berry phase is lost—the constant energy cuts of the photo-electron distribution are angle independent. However, the intensity of the photocurrent coming from the valence and conduction bands clearly shows the topological structure of the wavefunctions,


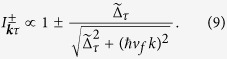


This is shown in Fig. 2 where the numerical simulation with the full time dependence of the pump and probe pulses are presented. The figure was obtained by fixing the chemical potential at a high energy (high doping) in order to show the photo-electron intensities corresponding to the valence and conduction bands in a wide energy range. The circularly polarized probe pulse acts at the centre of the pump pulse and its width in the time domain was chosen to be *δt*_probe_ = 50 fs to have a good energy resolution of the Floquet bands. The results clearly show that near the *K* point, the maximum intensity of the photo-electron distribution changes from the conduction to the valence band at the critical amplitude *A*_*c*_. This change is a consequence of the sign change of 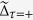 and is linked to a change of 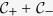.

To be more specific, we now present results for the case of graphene with realistic parameters. We used the experimentally observed value of the phase *β* = 0.4*π*, the chemical potential is set either at *μ* = 100 meV or *μ* = 0 meV, the frequency of the pump pulse is *ħ*Ω = 400 meV and the probe pulse is circularly polarized. These conditions generate small dichroism although its symmetry is different from that observed with *β* = 0: note that in cuts along *k*_*y*_ and in the absence of the pump pulse the lines with negative velocity in Fig. 3(a) are more intense around both the *K* and *K*′ points. The circularly polarized pump pulse with frequency Ω also opens gaps at the Floquet zone boundary (*ħ*Ω/2) that are detected by the ARPES spectrum^14^. The second order gap at the zone centre (zero energy) is not clearly observed due to the moderate amplitude of the pump and the width of the ARPES lines. However, the intensities of the lines corresponding to the conduction band show a marked different behavior at the two Dirac points as illustrated in Fig. 3(c). This behavior shows that the Berry curvature Ω_*kτ*_ defined above has the same sign for the two cones leading to a non-zero Chern number. This effect is also present when the chemical potential is fixed at *μ* = 0 eV as shown in Fig. 3(d), where the photoemission spectrum is presented along the *k*_*x*_ direction in order to disregard asymmetries due to the dichroism generated by the probe polarization.

In finite systems, the Floquet zone boundary gaps are also bridged by topologically protected edge states. In *k*-space, these edge states are confined arround the *K* and *K*′ points and their existence can be inferred by evaluating the Chern numbers with the Floquet bands^20^,^22^,^36^. The wavefunctions in the time domain clearly show that for those states bridging the zone-boundary gap the pseudospin oscillates with frequency Ω with its time average value on the *xy*-plane. The topological structure of these states, described by the above mentioned Chern numbers, is a real dynamical effect^37^. As the ARPES probe pulse averages on a time scale of the order of *δt*_probe_ ≫ 1/Ω, the photo-electrons can hardly carry some information on the topological nature of states at the zone-boundary gap.

It is worth mentioning that, as recently shown in ref. 38, the Chern number of a pure state (Slater determinant) cannot be changed by a unitary transformation. That is, the Chern of an initial state remains unaltered during the pump pulse. This fact of course does not prevent modifications of the band structure, the Floquet spectrum, and in particular the presence of the band inversion phenomena. In ARPES experiments with the appropriate energy and polarization, the interference of the dipole transitions allows for a clear identification of the different topological phases as revealed by the band inversion effect. With the help of a band structure model, that for graphene is well established, the ARPES intensity profiles allows to determine amplitude and phases of the wavefunctions for states close to the *K* and *K*′ points of the BZ and to reconstruct the Berry curvature around these hot spots.

The case of bilayer graphene, with a rather different band structure, is also interesting. The system has four *π*-bands, two of them, with parabolic dispersions, touch each other at the Dirac points and a gap can be opened and controlled by a perpendicular electric field. The other two bands lie at about 0.3 eV from the Dirac points. In the presence of the pump pulse these extra bands generate Floquet replicas that partially cover up the low energy ARPES spectrum making it much more intricate. Nevertheless, as the pump amplitude increases the topological transition evidenced by the band inversion phenomena can be clearly observed (see Supplementary Information).

In summary, we have shown that ARPES can give clear information on the topology of Floquet bands of graphene and graphene-like structures. This information is given by the intensity of the ARPES profiles of the bands close to the *K* and *K*′ points of the BZ. While in the topological trivial phase the intensities due to photo-electrons from the valence or conduction bands are similar at the two Dirac points, in the non-trivial phase the intensities of the valence and conduction bands are different and opposite at *K* and *K*′. This change signals a modification of the Berry curvature around these points with a consequent variation of the Chern numbers. To observe the effect the dipole transition matrix elements *ζ*_*x*_ and *ζ*_*y*_ should have a different phase *β*. It has been experimentally shown that in graphene *β* can be controlled with the photon energy of the probe pulse. This observation opens the road for a spectroscopic study of the topological properties of the *bulk* wavefunctions of these 2D materials.

## Additional Information

**How to cite this article**: Gavensky, L. P. *et al*. Photo-electrons unveil topological transitions in graphene-like systems. *Sci. Rep*. **6**, 36577; doi: 10.1038/srep36577 (2016).

**Publisher’s note:** Springer Nature remains neutral with regard to jurisdictional claims in published maps and institutional affiliations.

## Supplementary Material

Supplementary Information

## Figures and Tables

**Figure 1:**
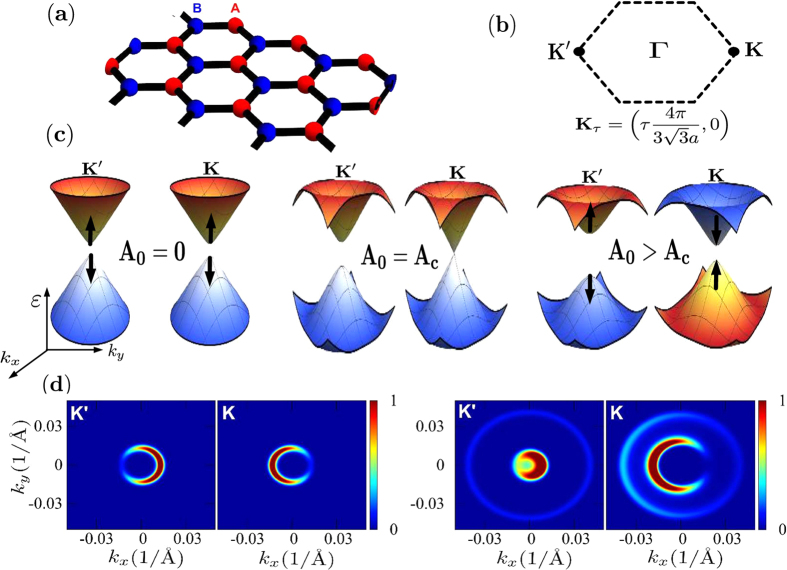
The hexagonal lattice with two sites (*A* and *B*) of the unit cell (**a**) and the corresponding Brillouin zone (**b**). In (**c**) the Floquet band structure near a Dirac point with the amplitude of the vector potential *A*_0_ = 0, *A*_*c*_ and *A*_0_ > *A*_*c*_ are shown; the color of the conduction and valence bands indicate the orientation of the pseudo-spin at the Dirac points. (**d**) The photoemission intensities at constant energy without the pump pulse (left) and with a high energy (*β* = 0, see text) linearly polarized probe pulse (right) show a dichroism characteristic of the chiral states; the only effect of the radiation in this configuration is to reveal the change of the constant energy surface at the *K* and *K*′ points and the appearance of Floquet replicas.

**Figure 2:**
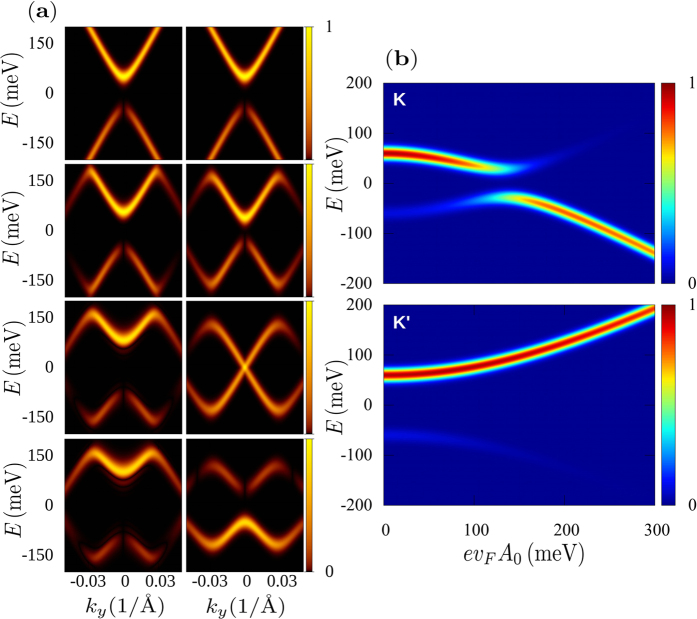
(**a**) ARPES intensity from states close to the *K*′ (left column) and *K* (right column) cones; the radiation intensity increases from top to bottom (field strength (*ev*_*F*_*A*_0_) at the peak of the pump pulse of 0 meV, 60 meV, 130 meV and 200 meV). These results correspond to a circularly polarized pump and probe pulses with *β* = *π*/2, the temporal duration of the former being of 350 fs and the latter of *δt*_probe_ = 50 fs. The chemical potential has been taken at 0.5 eV to appreciate the intensity changes of photo-electrons from both the valence and the conduction bands. The pump energy is *ħ*Ω = 400 meV. In (**b**) we show the intensities, from states with wavevector ***k*** slightly shifted from the Dirac points, as function of the radiation intensity. At the critical value of *ev*_*F*_*A*_0_ = 130 meV the ARPES intensity around *K* is transferred from the conduction to the valence band.

**Figure 3:**
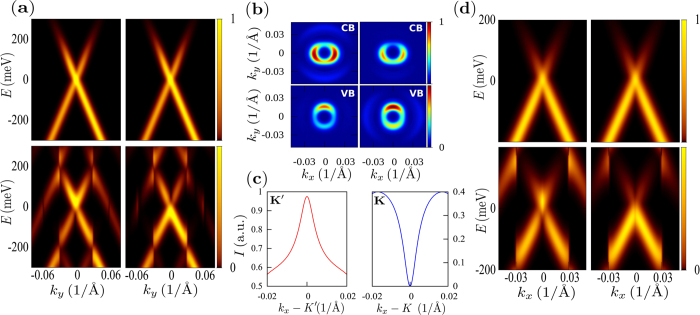
ARPES intensity for graphene (Δ = 0) and *β* = 0.4*π* corresponding to a probe photon of 20 eV.The pump and probe pulses are circularly polarized, the former with a time domain width of 250 fs and the latter with 20 fs. In all panels the left (right) column corresponds to the *K*′ (*K*) cone. (**a**) Cuts of the ARPES intensity along *k*_*y*_ for graphene in equilibrium (no pump pulse, upper panels) and irradiated graphene (wtih a pump pulse, lower panels) around the Dirac points. (**b**) Constant energy cuts at 100 meV (CB) and at −100 meV (VB). Note the small dichroism obtained with *β* = 0.4*π*. Contrary to the case of linearly polarized probe, now the dichroism around *K* and *K*′ has the same symmetry. (**c**) Intensity of the valence band photo-electrons along *k*_*x*_ from states close to each valley. The maximum at *K*′ and the minimum at *K* signals the non trivial topology of the Bloch wavefunctions of this band. (**d**) Cuts of the photoemission spectrum for graphene along *k*_*x*_ with chemical potential fixed at *μ* = 0 eV in equilibrium (upper panels) and irradiated (lower pannels) near–the Dirac points.
