# Supplementary material for: Photo-electrons unveil topological transitions in graphene-like systems
Source: Sci Rep. 2016 Nov 11;6:36577. doi: 10.1038/srep36577 (PMC5105119; doi:10.1038/srep36577)
Supplement: Supplementary Information [file srep36577-s1.pdf]

Supplementary information for "Photoelectrons unveil topological transitions in graphene-like systems"

Lucila Peralta Gavensky, Gonzalo Usaj, and C. A. Balseiro

Centro Atómico Bariloche and Instituto Balseiro,

Comisión Nacional de Energía Atómica, 8400 Bariloche, Argentina and

Consejo Nacional de Investigaciones Científicas y Técnicas (CONICET), Argentina

I. THE TIME EVOLUTION OPERATOR

The total Hamiltonian is written as $\mathcal{H}(t) = \sum_{\mathbf{k}\tau}\mathcal{H}_{\mathbf{k}\tau}(t)$ with

$$\mathcal{H}_{\mathbf{k}\tau}(t) = v_f\Pi^x_{\boldsymbol{k}\tau}(t)\sigma_x + v_f\Pi^y_{\boldsymbol{k}\tau}(t)\sigma_y + \Delta\sigma_z = \mathbf{d}_{\mathbf{k}\tau}(t)\cdot\boldsymbol{\sigma} \tag{1}$$

here $\mathbf{d}_{\mathbf{k}\tau}(t) = \Big(\tau v_f(\hbar k_x + eA_x(t)), \hbar v_f k_y + ev_f A_y(t), \Delta\Big)$, where the pump vector potential $\boldsymbol{A}(t) = \Re[\boldsymbol{A_0}(t)e^{i\Omega t}]$ has been introduced via minimal coupling ($\Pi^\nu_{\boldsymbol{k}\tau} = \hbar k_\nu + eA_\nu(t)$) with an envelope function $\boldsymbol{A_0}(t)$ and $\boldsymbol{\sigma} = (\sigma_x, \sigma_y, \sigma_z)$.

The time evolution operator from an initial time t_i to time t acting on a state with quantum numbers $\mathbf{k}$ and τ is

$$\mathcal{U}_{\mathbf{k}\tau}(t,t_i) = \mathcal{T}\Big[e^{-\frac{i}{\hbar}\int_{t_i}^{t}\boldsymbol{d}_{\mathbf{k}\tau}(t')\cdot\boldsymbol{\sigma}dt'}\Big], \tag{2}$$

where $\mathcal{T}$ is the time ordering operator. Using small time intervals δt the above integral is approximated as a sum

$$\mathcal{U}_{\mathbf{k}\tau}(t,t_i) = \mathcal{T}\Big[e^{-\frac{i}{\hbar}\sum_{n=1}^{N}\boldsymbol{d}_{\mathbf{k}\tau}(t_n)\cdot\boldsymbol{\sigma}\delta t}\Big] \approx \mathcal{T}\Big[\prod_n e^{-\frac{i}{\hbar}\boldsymbol{d}_{\mathbf{k}\tau}(t_n)\cdot\boldsymbol{\sigma}\delta t}\Big], \tag{3}$$

with $t_n = t_i + \frac{2n-1}{2}\delta t$. The last term in the above equation is obtained asuming that $[\mathcal{H}_{\mathbf{k}}(t_n), \mathcal{H}_{\mathbf{k}}(t_n + \delta t)] \approx 0$ for small enough δt. Using $[\boldsymbol{\sigma}\cdot\hat{\boldsymbol{d}}_{\boldsymbol{k}\tau}(t_n)]^{2n} = \mathbf{1}$ and $[\boldsymbol{\sigma}\cdot\hat{\boldsymbol{d}}_{\boldsymbol{k}\tau}(t_n)]^{2n+1} = \boldsymbol{\sigma}\cdot\hat{\boldsymbol{d}}_{\boldsymbol{k}\tau}(t_n)$, with $\hat{\boldsymbol{d}}_{\boldsymbol{k}\tau} = \boldsymbol{d}_{\boldsymbol{k}\tau}/|\boldsymbol{d}_{\boldsymbol{k}\tau}|$, the time evolution operator can be written as

$$\mathcal{U}_{\mathbf{k}\tau}(t,t_i) = \mathcal{T}\Big[\prod_n\Big\{\cos\Big(|\mathbf{d}_{\mathbf{k}\tau}(t_n)|\frac{\delta t}{\hbar}\Big)\mathbf{1} - i\sin\Big(|\mathbf{d}_{\mathbf{k}\tau}(t_n)|\frac{\delta t}{\hbar}\Big)\boldsymbol{\sigma}\cdot\hat{\boldsymbol{d}}_{\mathbf{k}\tau}(t_n)\Big\}\Big]. \tag{4}$$

To illustrate the effect of the pump pulse on an unperturbed graphene wavefunction $|\Phi^\gamma_{\boldsymbol{k}\tau}\rangle$ where $\gamma = \pm$ stands for a state in the valence and conduction band respectively, we calculate the probability $P_{\boldsymbol{k}}(t) = |\langle\Phi^{\gamma'}_{\boldsymbol{k}\tau}|\mathcal{U}_{\mathbf{k}\tau}(t,t_i)|\Phi^\gamma_{\boldsymbol{k}\tau}\rangle|^2$ of finding a final state $|\Phi^{\gamma'}_{\boldsymbol{k}\tau}\rangle$ at time t with an initial time t_i preceding the pumping.

According to the Floquet theorem when the system is perturbed by circularly polarized radiation of frequency Ω, the Floquet spectrum shows gaps at the Floquet zone centre, with zero energy, and at the Floquet zone-boundary of energy $\hbar\Omega/2$. These energies correspond to wavectors $k = 0$ and $k = k_0 = \Omega/2v_f$. Fig. 1 shows that even for very short pump pulses, the evolution of the wave functions with $k = 0$ and $k = k_0$ considerably differs from those with other values of k away from any anticrossing of the spectrum. In fact for $k = 0, k_0$ the system is in a resonant condition, with the pseudospin oscillating between the *up* and *down* states with a dominant frequency ω given by the corresponding Floquet gap. For other values of k, an out of resonance condition, the amplitude of the oscillations decreases and its main frequency is the frequency Ω of the pump.

II. DIPOLAR MATRIX ELEMENTS

The eigenfunctions of the Hamiltonian given by Eq. (1) with $\Delta \neq 0$ are given by

$$\begin{aligned}
|\Psi^{+}_{\boldsymbol{k}\tau}\rangle &= \cos\left(\frac{\phi_{\boldsymbol{k}\tau}}{2}\right)|\boldsymbol{k},A\rangle + \sin\left(\frac{\phi_{\boldsymbol{k}\tau}}{2}\right)e^{i\tau\theta_{\boldsymbol{k}}}|\boldsymbol{k},B\rangle \\
|\Psi^{-}_{\boldsymbol{k}\tau}\rangle &= \sin\left(\frac{\phi_{\boldsymbol{k}\tau}}{2}\right)|\boldsymbol{k},A\rangle - \cos\left(\frac{\phi_{\boldsymbol{k}\tau}}{2}\right)e^{i\tau\theta_{\boldsymbol{k}}}|\boldsymbol{k},B\rangle,
\end{aligned} \tag{5}$$

where $|\boldsymbol{k},A\rangle$ and $|\boldsymbol{k},B\rangle$ are the Bloch wavefunctions of the A and B sublattice respectively and the $\pm$ index refers to the conduction and valence bands, $\theta_{\boldsymbol{k}}$ is the angle formed by $\boldsymbol{k}$ and the x-axis, $\cos\left(\frac{\phi_{\boldsymbol{k}\tau}}{2}\right) = \tau\hbar v_f|\boldsymbol{k}|/\sqrt{(\hbar v_f k)^2 + (\varepsilon_+ - \Delta)^2}$ and $\sin\left(\frac{\phi_{\boldsymbol{k}\tau}}{2}\right) = (\varepsilon_+ - \Delta)/\sqrt{(\hbar v_f k)^2 + (\varepsilon_+ - \Delta)^2}$ with $\varepsilon_+ = \sqrt{\Delta^2 + (\hbar v_f k)^2}$.

The dipolar matrix elements $\langle f|\boldsymbol{P}_A\cdot\boldsymbol{p}|\Psi^{\pm}_{\boldsymbol{k}\tau}\rangle$ are given in terms of $\zeta_x = \langle f|p_x|\boldsymbol{k}A\rangle = \langle f|p_x|\boldsymbol{k}B\rangle$ and $\zeta_y = \langle f|p_y|\boldsymbol{k}A\rangle = -\langle f|p_y|\boldsymbol{k}B\rangle$, where the relative signs are due to the symmetries of the graphene lattice. The vector potential describing the probe (ARPES) pulse is $\boldsymbol{A}_{pr}(t) = A_{pr}(t)\Re[e^{i\omega t}\boldsymbol{P}_A]$ where $\boldsymbol{P}_A = \cos(\chi)\hat{\boldsymbol{x}} - i\sin(\chi)\hat{\boldsymbol{y}}$ and defining $\zeta_y/\zeta_x = \lambda e^{i\beta}$ the matrix elements are

$$\begin{aligned}
M^{+}_{\boldsymbol{k}\tau} &\propto \cos(\chi)\Big\{\cos\left(\frac{\phi_{\boldsymbol{k}\tau}}{2}\right) + \sin\left(\frac{\phi_{\boldsymbol{k}\tau}}{2}\right)e^{i\tau\theta_{\boldsymbol{k}}}\Big\} - i\sin(\chi)\lambda e^{i\beta}\Big\{\cos\left(\frac{\phi_{\boldsymbol{k}\tau}}{2}\right) - \sin\left(\frac{\phi_{\boldsymbol{k}\tau}}{2}\right)e^{i\tau\theta_{\boldsymbol{k}}}\Big\} \\
M^{-}_{\boldsymbol{k}\tau} &\propto \cos(\chi)\Big\{\sin\left(\frac{\phi_{\boldsymbol{k}\tau}}{2}\right) - \cos\left(\frac{\phi_{\boldsymbol{k}\tau}}{2}\right)e^{i\tau\theta_{\boldsymbol{k}}}\Big\} - i\sin(\chi)\lambda e^{i\beta}\Big\{\sin\left(\frac{\phi_{\boldsymbol{k}\tau}}{2}\right) + \cos\left(\frac{\phi_{\boldsymbol{k}\tau}}{2}\right)e^{i\tau\theta_{\boldsymbol{k}}}\Big\},
\end{aligned} \tag{6}$$

The ratio $\zeta_y/\zeta_x = \lambda e^{i\beta}$ depends on the x-ray energies of the ARPES excitation, experimental values for λ and β are given in Ref. [1]. It is important to note that for $\beta = \frac{\pi}{2}$ and $\lambda = 1$, admitting the possibility of photoemitting electrons with a probe pulse with circular polarization ($\chi = \pm\frac{\pi}{4}$), a selective projection of the pseudospin along the z axis is achievable. This means that it is plausible to generate a photoelectron current with entirely A or B character, depending on whether the probe polarization is right or left, respectively. These matrix elements are used for the numerical calculation of the photoelectron intensity. The numerical results with the full time dependence of the driving pump near the Dirac cones can be interpreted in terms of the approximate expression

$$\begin{aligned}
I^{\pm}_{\boldsymbol{k}} \propto |M^{\pm}_{\boldsymbol{k}\tau}|^2 = \cos^2(\chi) + \lambda^2\sin^2(\chi) \pm \Big\{&\sin(\widetilde{\phi}_{\boldsymbol{k}\tau})\cos(\theta_{\boldsymbol{k}})[\cos^2(\chi) - \lambda^2\sin^2(\chi)] \\
&+\lambda\sin(2\chi)[\sin(\beta)\cos(\widetilde{\phi}_{\boldsymbol{k}\tau}) - \cos(\beta)\sin(\widetilde{\phi}_{\boldsymbol{k}\tau})\sin(\tau\theta_{\boldsymbol{k}})]\Big\},
\end{aligned} \tag{7}$$

here $\cos(\widetilde{\phi}_{\boldsymbol{k}\tau}) = \widetilde{\Delta}/\sqrt{\widetilde{\Delta}^2 + (\hbar v_f k)^2}$ and $\sin(\widetilde{\phi}_{\boldsymbol{k}\tau}) = \tau\hbar v_f|\boldsymbol{k}|/\sqrt{\widetilde{\Delta}^2 + (\hbar v_f k)^2}$. The mass term $\widetilde{\Delta}$ is renormalized by the presence of the circular electromagnetic driving.

The dichroism factor $D_{\pm}(\boldsymbol{k})$ is defined as $D_{\pm}(\boldsymbol{k}) = \{\text{Max}[I^{\pm}_{\boldsymbol{k}}(\theta_{\boldsymbol{k}})] - \text{Min}[I^{\pm}_{\boldsymbol{k}}(\theta_{\boldsymbol{k}})]\}/\{\text{Max}[I^{\pm}_{\boldsymbol{k}}(\theta_{\boldsymbol{k}})] + \text{Min}[I^{\pm}_{\boldsymbol{k}}(\theta_{\boldsymbol{k}})]\}$. For $\beta = \pi/2$ this gives

$$D^{\pm}(\boldsymbol{k}) = \sin(\phi_{\boldsymbol{k}\tau})\cos(2\chi)/(1 \pm \cos(\phi_{\boldsymbol{k}\tau})\sin(2\chi)) \tag{8}$$

III. ARPES IN BILAYER GRAPHENE

In the Bernal structure the unit cell of the Bilayer Graphene has four C atoms. Consequently there are four π-bands, two of them with opposite parabolic dispersions touch each other at the Dirac points. The other two bands are shifted by $\approx 0.3eV$. These four low energy bands are described by the Hamiltonian $H = H_1 + H_2 + H_{12}$ where the first terms describes the electronic structure of two isolated graphene sheets and the last term the interplane coupling

$$\begin{aligned} H_i = V(-1)^{i-1} \sum_{\mathbf{k},\sigma} [a^{\dagger}_{i,\mathbf{k},\sigma} a_{i,\mathbf{k},\sigma} + b^{\dagger}_{i,\mathbf{k},\sigma} b_{i,\mathbf{k},\sigma} \\ -t(\phi(\mathbf{k}) a^{\dagger}_{i,\mathbf{k},\sigma} b_{i,\mathbf{k},\sigma} + \phi^*(\mathbf{k}) b^{\dagger}_{i,\mathbf{k},\sigma} a_{i,\mathbf{k},\sigma})] \end{aligned} \tag{9}$$

with $i = 1, 2$, $a_{i,\mathbf{k},\sigma}$ and $b_{i,\mathbf{k},\sigma}$ destroy electrons with wavector $\mathbf{k}$ and spin σ in sublattices A and B of the ith plane respectively and we have included an electric field perpendicular to the BLG plane described by V. The matrix element t corresponds to the intraplane hopping and

$$\phi(\mathbf{k}) = e^{iak_y} \left[1 + 2e^{-i\frac{3a}{2}k_y} \cos\left(\frac{a\sqrt{3}}{2} k_x \right) \right] \tag{10}$$

with $a = 1.42$ Å the carbon-carbon distance.

The interplane coupling is described by:

$$H_{12} = \sum_{\mathbf{k},\sigma} t_{\perp} (a^{\dagger}_{1\mathbf{k},\sigma} b_{2,\mathbf{k},\sigma} + b^{\dagger}_{2,\mathbf{k},\sigma} a_{1,\mathbf{k},\sigma}) \tag{11}$$

For each value of the wave-number $\mathbf{k}$ we have a $4X4$ Hamiltonian $H_{\mathbf{k}}$ given by

$$H_{\mathbf{k}} = \begin{vmatrix} V & -t\phi(\mathbf{k}) & 0 & t_{\perp} \\ -t\phi^*(\mathbf{k}) & V & 0 & 0 \\ 0 & 0 & -V & -t\phi(\mathbf{k}) \\ t_{\perp} & 0 & -t\phi^*(\mathbf{k}) & -V \end{vmatrix} \tag{12}$$

with wavector $[u^m_{A1}(\mathbf{k}), u^m_{B1}(\mathbf{k}), u^m_{A2}(\mathbf{k}), u^m_{B2}(\mathbf{k})]^T$ and eigenvalues $\varepsilon_m(\mathbf{k})$. Linearizing around the Dirac cones K_{τ} and coupling the crystal momentum with radiation via Peierls substitution one

can readily obtain a time dependent $H_{\boldsymbol{k}\tau}(t)$. The complete evolution of the wavefunction during the driving pulse is obtained numerically by means of the evolution operator. In this case, this time dependent propagator is computed by an exact diagonalization of the hamiltonian at each instant of time:

$$\mathcal{U}_{\boldsymbol{k}\tau} = \mathcal{T}\Big[\prod_n \sum_m e^{-i\varepsilon_{\boldsymbol{k}m}(t_n)\frac{\delta t}{\hbar}} |\Psi^m_{\boldsymbol{k}\tau}(t_n)\rangle\langle\Psi^m_{\boldsymbol{k}\tau}(t_n)|\Big] = \mathcal{T}\Big[\prod_n \mathbb{P}_{\boldsymbol{k}\tau}(t_n)\Big], \tag{13}$$

where $\mathbb{P}_{\boldsymbol{k}\tau}(t_n) = \sum_m e^{-i\varepsilon_{\boldsymbol{k}m}(t_n)\frac{\delta t}{\hbar}} |\Psi^m_{\boldsymbol{k}\tau}(t_n)\rangle\langle\Psi^m_{\boldsymbol{k}\tau}(t_n)|$ and m is the band index. The dipolar matrix elements at each valley take the form

$$M^{\tau}_{\boldsymbol{k}m} \propto \cos(\chi)[u^m_{A1}(\mathbf{k})+u^m_{B1}(\mathbf{k})+u^m_{A2}(\mathbf{k})+u^m_{B2}(\mathbf{k})]-i\sin(\chi)\lambda e^{i\beta}[u^m_{A1}(\mathbf{k})-u^m_{B1}(\mathbf{k})+u^m_{A2}(\mathbf{k})-u^m_{B2}(\mathbf{k})]. \tag{14}$$

Taking the limit of $\beta = \frac{\pi}{2}$, $\lambda = 1$ and circular polarization $\chi = \frac{\pi}{4}$ the generated photocurrent has only $\mathcal{A}_1$ and $\mathcal{A}_2$ character. By changing the quirality of the probe polarization to $\chi = \frac{7\pi}{4}$ the radiation field couples with $\mathcal{B}_1$ and $\mathcal{B}_2$ sublattices.

The low energy excitations with crystal momentum arround the K and K' points of the BZ can be described by an effective two band Hamitonian, obtained by eliminating the bands that are shifted from the Fermi energy by $t_\perp$ by means of a canonical transformation. In the base of the $A2$ and $B1$ orbitals, the effective Hamiltonian for a given wavevector $\mathbf{k}$ takes the form:

$$H_{\boldsymbol{k}\tau} = \boldsymbol{h}_\tau(\boldsymbol{k})\cdot\boldsymbol{\sigma} \tag{15}$$

where $\boldsymbol{\sigma}$ are the Pauli matrices and

$$\begin{aligned} h_x &= \alpha\left(k_x^2 - k_y^2\right), \\ h_y &= 2\alpha\tau k_x k_y, \\ h_z &= -V, \end{aligned} \tag{16}$$

with $\alpha = \frac{9}{4}\frac{(ta)^2}{t_\perp} = \frac{(\hbar v_f)^2}{t_\perp}$. This two band effective problem is similar to the graphene with mass model, with the advantage of having the possibility to regulate at will the electric field in order to change the parameter V. The quadratic (instead of linear) dispersion of these low energy bands is responsable for a coupling of higher order with Floquet replicas, with a lowest order modification of the bias given by

$$\tilde{V}_\tau = V + \tau\frac{(ev_f A_0)^4}{t_\perp^2(2\hbar\Omega + \tau V)}. \tag{17}$$

The effect of a renormalization of V when the system is irradiated generates a closing gap in the cuasi-energy spectrum at one Dirac point and a corresponding opening at the other, making it plausible to detect the band inversion phenomena, as shown in Fig. 2. The frequency of the pump pulse was set at $\hbar\Omega = 0.5\,\mathrm{eV}$ in order to neglect the influence of replicas from the high energy bands near the Dirac cones. The ARPES instensity of the effective bilayer two band model is also shown in Fig. 3 for each valley. In this case the incident photon radiation was set at $\hbar\Omega = 0.2\,\mathrm{eV}$.

---

[1] Y. Liu, G. Bian, T. Miller, and T.-C. Chiang, "Visualizing electronic chirality and berry phases in graphene systems using photoemission with circularly polarized light," Phys. Rev. Lett. **107** (2011), 10.1103/physrevlett.107.166803.

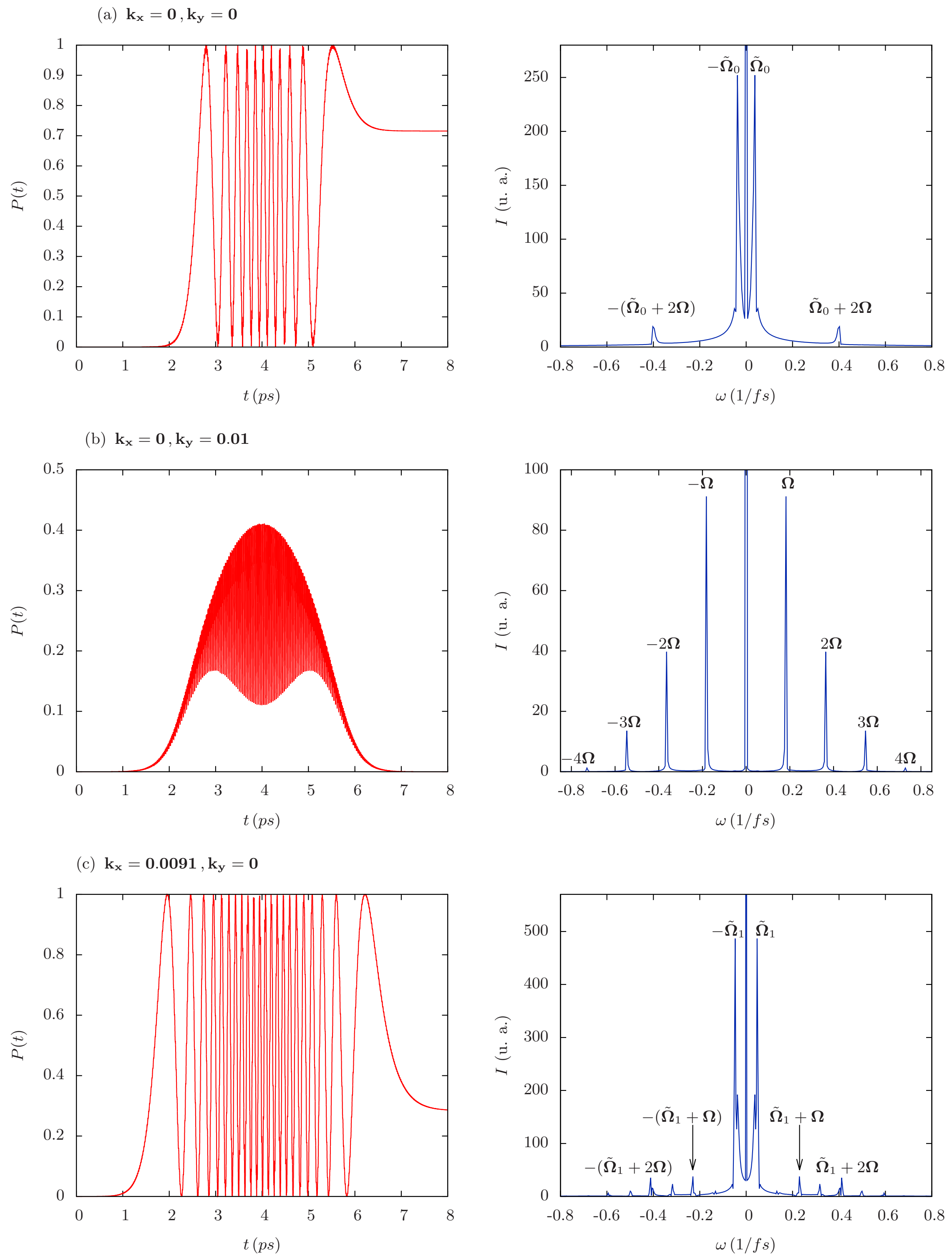

FIG. 1. Probability $P_{\boldsymbol{k}}(t) = |\langle \Phi^+_{\boldsymbol{k}\tau}|\mathcal{U}_{\mathbf{k}\tau}(t,t_i)|\Phi^-_{\boldsymbol{k}\tau}\rangle|^2$ of finding a final state $|\Phi^+_{\boldsymbol{k}\tau}\rangle$ at the conduction band at time t with an initial state $|\Phi^-_{\boldsymbol{k}\tau}\rangle$ at the valence band at time t_i preceding the pumping for crystal momentum (left pannels) (a) at the Dirac cone K, (b) away from any anticrossing and (c) at $k_0 = \frac{\Omega}{2v_f}$ with their corresponding fourier transform (right pannels).

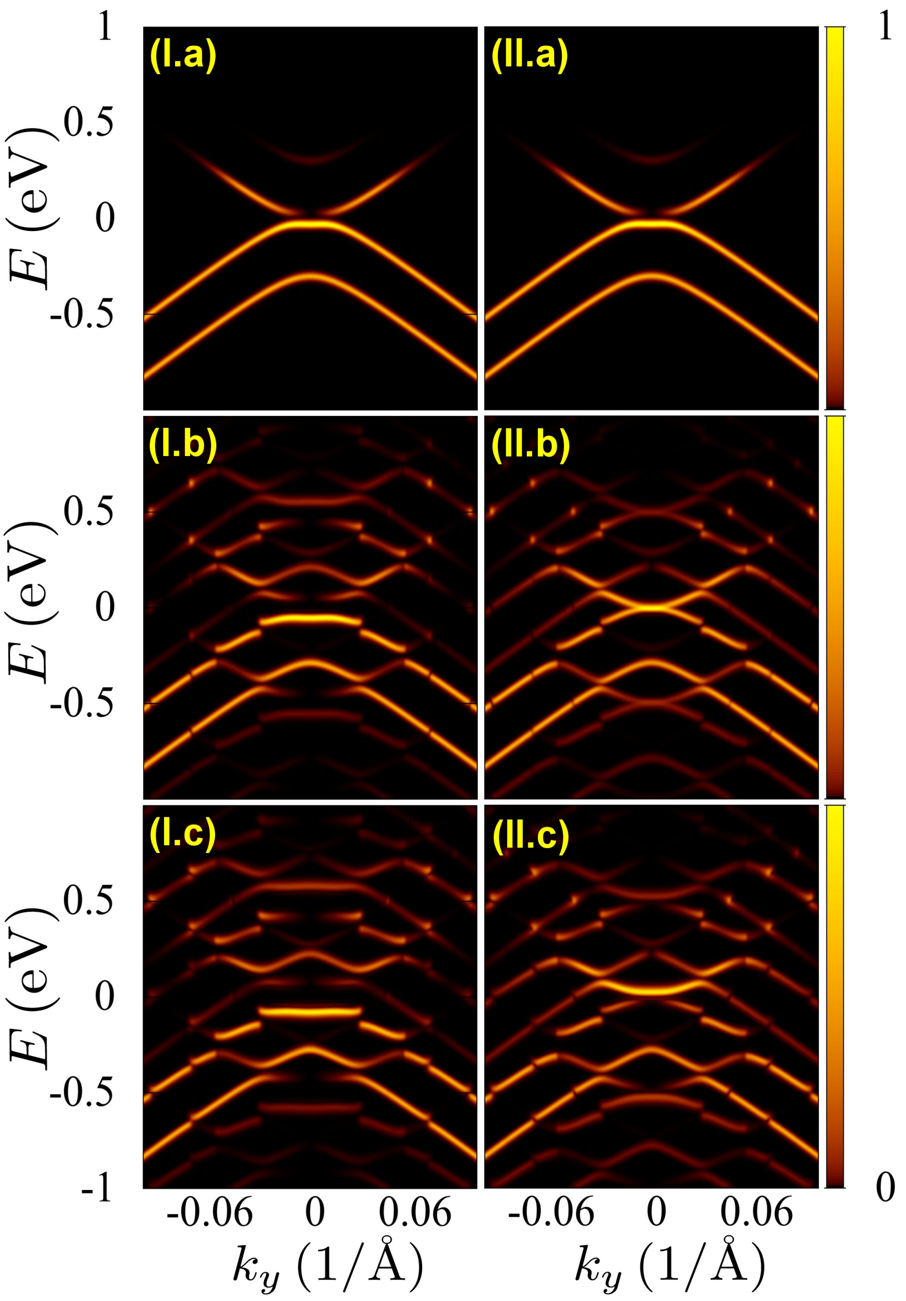

FIG. 2. ARPES intensity of bilayer graphene from states close to K (left column) and K' (right column) Dirac cones; the radiation intensity increases from top to bottom. These results correspond to a circularly polarized pump and probe pulses with $\beta = \pi/2$. The chemical potential has been taken at 0.3 eV.

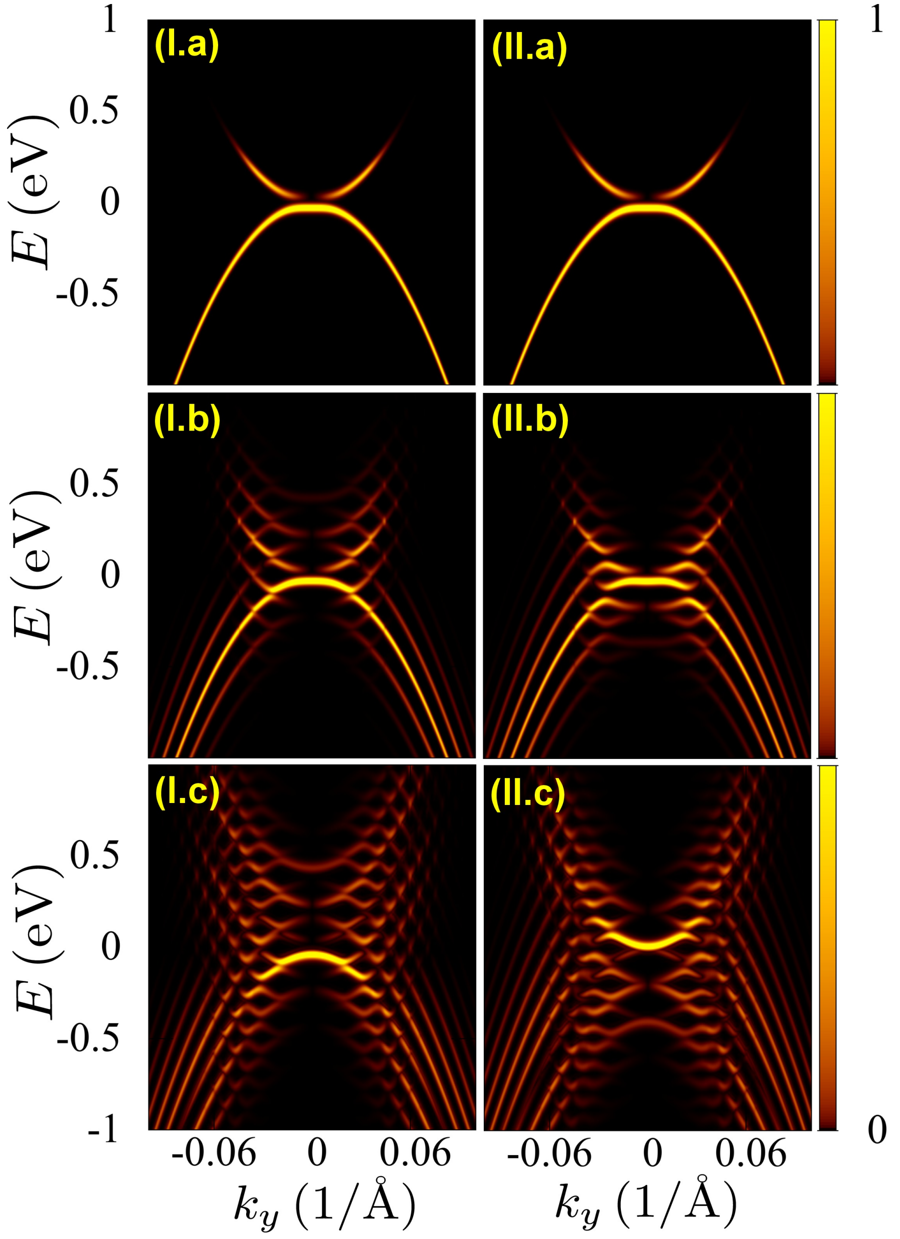

FIG. 3. ARPES intensity of the effective bilayer two band model from states close to K (left column) and K' (right column) Dirac cones; the radiation intensity increases from top to bottom. These results correspond to a circularly polarized pump and probe pulses with $\beta = \pi/2$. The chemical potential has been taken at 0.3 eV.
